# Use of Statistical Design Strategies to Produce Biodegradable and Eco-Friendly Films from Cashew Gum Polysaccharide and Polyvinyl Alcohol

**DOI:** 10.3390/ma12071149

**Published:** 2019-04-09

**Authors:** Maurício V. Cruz, Marcos A. Pereira-Júnior, Karla A. Batista, Kátia F. Fernandes

**Affiliations:** 1 Departamento de Áreas Acadêmicas, Instituto Federal de Educação, Ciência e Tecnologia de Goiás, Campus Uruaçu, 76400-000 Uruaçu-GO, Brazil; mauricio.vicente@ifg.edu.br; 2 Laboratório de Química de Polímeros, Departamento de Bioquímica e Biologia Molecular, Instituto de Ciências Biológicas 2, Campus Samambaia, Universidade Federal de Goiás, 74690-900 Goiânia-GO, Brazil; marcos.apjunior@gmail.com; 3 Departamento de Áreas Acadêmicas, Instituto Federal de Educação, Ciência e Tecnologia de Goiás, Campus Goiânia Oeste, 74270-040 Goiânia-GO, Brazil

**Keywords:** CGP, biopolymer, factorial design, green technology

## Abstract

This work reports the production and characterization of biodegradable and eco-friendly films based on cashew gum polysaccharide (CGP) and polyvinyl alcohol (PVA), using the statistical design strategy. Results show that CGP/PVA films are pH stimuli-responsive, allowing their use in a magnitude of biotechnological applications. The morphological and dimensional characterization evidences a positive influence of polymers in the dimensional properties. In addition, the microstructural analysis shows that films have different morphologies depending on the content of polymers and oxidant agent. On the other hand, the thickness and light transmission values are positively influenced by CGP and PVA and negatively influenced by NaIO_4_. Results from mechanical properties show that the traction force is positively influenced by NaIO_4_, while the elongation is only affected by the PVA concentration. In summary, considering the morphological, optical and mechanical properties of the CGP/PVA films it is possible to suggest their utilization in different fields as promising packaging materials or matrices for immobilization and/or encapsulation of biomolecules.

## 1. Introduction

Plastics have become indispensable to the modern society. The reasons for their massive use are associated with their excellent properties as water and microorganism barriers, high mechanical resistance and low production cost. Their use is substantial in the package industrial sector, where they are employed in the food, pharmaceutical and cosmetic areas [1,2,3]. Consequently, a large amount of post-consumer plastics waste is daily generated. 

Even with their expressive economic advantages, the large amount of plastic wastes produced and the long time required to decompose these materials through physical, chemical and biological processes caused plastics to be associated with critical environmental issues, related to their non-biodegradability [2]. In this sense, the search for biodegradable materials from renewable sources to total or partially replace plastics is a trend of the market sustained by consumer demand. 

In the last decades, biodegradable films materials based on carbohydrates, proteins and lipids have been largely studied especially due to their accessibility and lower production costs. However, biopolymer films could have some usage limitations related to their hydrophilic characteristics and barrier properties [4]. In this sense, several researches have been performed with the aim to study the physicochemical features of hybrid biopolymers composed by natural and synthetic molecules. Hybrid films produced by blending two or more polymers are referred to present improved or modified mechanical and barrier properties, as well as biodegradability and biocompatibility [5,6,7].

In general, the production of hybrid biopolymers is based on the introduction of reactive groups in the polymer molecules or the use of crosslinking agents. Periodate oxidation is a simple and effective method for the introduction of reactive groups in polysaccharides and hydroxyl-containing molecules. For polysaccharides, the attack by the periodate ion (IO_4_^−^) leads to the formation of aldehyde groups, while in hydroxyl-containing molecules, the periodate ions oxidize the hydroxyls to carboxylic acids. These reactive sites are frequently used as a chemical anchor for reactions with nucleophilic molecules, allowing the production of materials with different properties and applications [8,9]. 

Several biodegradable new hybrid materials based on carbohydrates have been reported in the literature [10,11,12,13]. Among them, materials based on the blend of cashew gum polysaccharide (CGP) and polyvinyl alcohol (PVA) has shown very good properties regarding their biodegradability. In a previous work, a set of CGP/PVA films with mechanical properties superior and/or similar to a commercial membrane of bovine origin were synthesized [14]. In this study, a stimuli-responsive, biodegradable and bioactive film was produced by blending cashew gum polysaccharide (CGP) and polyvinyl alcohol (PVA). This CGP/PVA film presented malleability and mechanical properties enabling an easy handling. Wetting the film changed the optical properties from opacity to levels of transparency higher than 70% and resulted in up to 2-fold increase in its superficial area. Different swelling indexes were obtained varying the pH of solvent, which allows classifying the CGP/PVA film as pH-sensitive material. The bioactivity was achieved through the covalent immobilization of papain, which remained active after the storage of CGP/PVA-papain film for 24 h in the presence of a buffer or in a dry form. These results evidenced that CGP/PVA-papain film is a very promising material for biomedical applications. Moreira et al. [15] also synthesized a CGP/PVA film, which was functionalized by immobilization of trypsin onto its surface and this bioactive film was proposed to be used as a bioactive wound healing coverage. 

All these films were prepared by changing the concentration and/or components in a minor proportion from a standard formulation. However, according to Silva et al. [16], the main mechanism of reaction between the polymers in the CGP/PVA films involve the oxidized carbonyl groups in PVA and hydroxyl groups present in C2 and C3 of CGP mediated by sodium periodate [10]. These reactive groups are responsible for the formation of the linkages that will compose the network of the film. In this sense, changes in the amount of the available reactive groups will result in changes in the film properties and morphology. 

Considering the promising features of the CGP/PVA materials, it was clear the necessity of understanding the relationship among its components in order to advance in the product development. An interesting approach to study the effect of the constituents on the biotechnological characteristics of CGP/PVA matrixes is to use factorial designs. However, it is often too costly and time-consuming to perform a full factorial experiment. So, it is better to use a fractional factorial design, which is a fraction of a full factorial design. The main advantage of fractional factorial designs is that they enable us to build statistical models with a small number of runs. When this fraction is accurately selected, the resulting design can estimate the maximum number of factorial effects of interest with maximum precision [11,12].

Therefore, in the present work a 3^3-1^ fractional factorial design was applied to study the effects of CGP, PVA and sodium periodate concentrations on the biotechnological properties of CGP/PVA films. The produced films were analyzed regarding their morphological, mechanical and optical properties. 

## 2. Materials and Methods 

### 2.1. Cashew Gum Polysaccharide Extraction

The polysaccharide extraction was performed according to methodology described by Cruz et al. [17]. Briefly, the nodules of cashew gum were ground and solubilized in water in a proportion of 20% (w/v). The suspension was filtered and the polysaccharide (CGP) was precipitated with cold ethanol (1:3). The CGP was separated by centrifugation and dried at room temperature. All samples of cashew gum used in this work were collected from *Anacardium occidentale* trees at Aquirás (Ceará, Brazil).

### 2.2. CGP/PVA Films Production

The CGP/PVA films were prepared according to methodology described by Cruz et al. [17]. In order to evaluate the effects of CGP, PVA and NaIO_4_ concentrations on the biotechnological properties of the formed films, a 3^3-1^ fractional factorial design was used (Table 1). The film-forming solutions were prepared by mixing 50 mL of each polymer (CGP and PVA) with 5 mL of oxidant agent (NaIO_4_), 10 mL of 1.0 mol L^−1^ phosphoric acid solution (catalyst) and 15 mg of mannitol. The solutions were cast in molds and the solvent was evaporated at room temperature. The dried films were peeled from the casting mold and exhaustively washed with distilled water to completely remove unreacted materials (wash water). Then, films were dried at room temperature and stored in plastic vials. Samples of CGP/PVA films were chosen considering film uniformity and the absence of defects (i.e., air bubbles, holes, tears, flaws, etc.).

### 2.3. Released Carbohydrates

The wash water collected as described above was used to determine the carbohydrate content according to the method described by Dubois et al. [18] The reaction was performed by mixing 1.0 mL of sample with 1.0 mL of 80% phenol solution (w/w) and 5.0 mL of concentrated sulfuric acid. After cooling, absorbance was measured at 490 nm in an ultraviolet-visible (UV-Vis) spectrophotometer (SP-220, Biospectro, Curitiba, Brazil). The total carbohydrate content was calculated from a standard curve using cashew gum polysaccharide as standard (*r*^2^ = 0.995).

### 2.4. Swelling Index

For the determination of the swelling index, CGP/PVA films were cut into 1 cm^2^ strips, weighted, immersed in 5 mL of different solutions and incubated at 25 °C. The swelling behavior was tested using the following solutions: (1) Ultrapure water (milli-Q); (2) 0.1 mol L^−1^ sodium acetate buffer pH 4.5; (3) 0.1 mol L^−1^ glycine buffer pH 9.5 [16,17].

The changes in the water sorption capacity of the films were monitored at regular time intervals, until reaching the swelling stabilization. Before measuring the swollen weight, films were wiped with absorbent paper to remove water on the film surface. The swelling index of each sample at time *t* was obtained using Equation (1):

(1) $$Swelling\;index=\left(W_{t}-W_{0}\right)/W_{0}$$

where, *W_t_* is the swollen weight of the CGP/PVA film at time *t* and *W_0_* is the weight of the dried sample. 

### 2.5. Dimensional Changes

Aiming to investigate the magnitude of dimensional changes during swelling, CGP/PVA films were cut into 1 cm^2^ strips and the three-dimensional area after 10 min of swelling was measured using a digital micrometer (MDC-SX/25mm, Mitutoyo Corp., Tokyo, Japan).

### 2.6. Microstructural Analysis

In order to evaluate the effect of pH on the microstructure of CGP/PVA matrixes, the swollen films were examined by the scanning electron microscopy (SEM). After swollen, the CGP/PVA films were lyophilized and the SEM analysis were performed in a JEOL JSM 6610 scanning electron microscope (Tokyo, Japan), using a secondary electron detector with 15 kV of acceleration. These experiments were performed in the Laboratory Laboratório Multiusuário de Microscopia de Alta Resolução (LabMic) at the Federal University of Goias, GO, Brazil. 

### 2.7. Light Transmission Measurement

Light transmission measurements were performed according to the methodology described by Wang and Xiong [19], using wet and dried CGP/PVA films. CGP/PVA strips with 4 × 2 cm^2^ were analyzed in a UV-Vis Spectrophotometer (Biospectro SP-220, Curitiba, Brazil), in the wavelength ranging from 400 to750 nm, with a step size of 5 nm. The mean of transmittance values in the visible region was used as data for the 3^3-1^ fractional factorial design.

### 2.8. Thickness

The thickness of the CGP/PVA films were determined as described by Cao et al. [20], using a digital micrometer (Mitutoyo Corp., Tokyo, Japan) with an accuracy of 0.001 mm. The films from the 3^3-1^ fractional factorial design were submitted to 10 random measurements carried out on the cross-section of the films. 

### 2.9. Mechanical Properties

The traction force and the elongation properties of the produced CGP/PVA films were determined in a texture analyzer (Lloyd Instruments/Ametek TA1, Largo, FL, USA), following the ASTM standard method D882-02 [21]. Before determination, samples had their thickness measured (10 random points) and slices of 15 mm × 90 mm were conditioned at 50% relative humidity (RH) for 24 h (25 °C) in a desiccator containing a saturated solution of potassium carbonate. Tests were conducted using a cross read speed of 300 mm min^−1^ and an initial grid separation of 50 mm. 

### 2.10. Statistical Analysis

All analyses were performed in triplicate with repetitions, and the mean values were reported. Statistica Software (Statistica 7.0, Stat Soft Inc., Tulsa, OK, USA) was used to perform the analysis of the data from the 3^3-1^ fractional factorial design. The models were simplified by dropping terms that have no statistical significance (*p* = 0.05).

## 3. Results

### 3.1. Production of CGP/PVA films

In order to evaluate the retention of CGP in the CGP/PVA network, the content of unbound carbohydrates was determined in the washing waters (Table 2). The highest values of CGP releasing were observed in the CGP/PVA from Run 3 (41.2 mg mL^−1^) and Run 6 (43.2 mg mL^−1^). On the other hand, films produced using 6% PVA, 1% CGP and 0.75 mol L^−1^ NaIO_4_ showed the lowest values of released carbohydrates (11.2 mg mL^−1^), suggesting that the CGP retention could be dependent of the PVA concentration in the formulation. 

**Figure 1:**
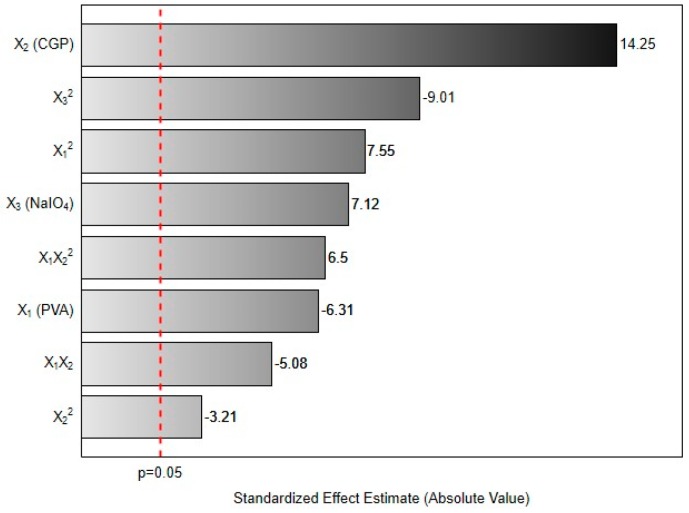
Pareto chart of the main effects obtained from 3^3-1^ fractional factorial design for released carbohydrates determination.

Results from the multivariate analysis evidenced that all factors significantly affected the response. As can be seen in Figure 1, the linear terms for CGP (X_2_) and NaIO_4_ (X_3_) concentrations, the quadratic term for PVA concentration (X_1_^2^) and the interaction factor for X_1_X_2_^2^ had a positive effect on the content of released carbohydrates. This result indicates that increases in these factors will increase the amount of CGP released from the formed CGP/PVA films. On the other hand, the linear term for PVA (X_1_), quadratic terms for CGP (X_2_^2^) and NaIO_4_ (X_3_^2^) concentrations and the interaction factor for X_1_X_2_ negatively affected the response.

Analyzing these effects is possible to understand the relationship among the reactants in the formation of the film network. The pronounced positive effect of CGP (14.2) is a predictable result, since its excess would result in residual unbound polysaccharides, contributing to increase the released carbohydrate content. Alternatively, the expressive negative linear effect of PVA (−6.31) evidences the function of this polymer in the formation of the CGP/PVA film. The increase in PVA concentration leads to a higher CGP retention in the network. This inference is confirmed by the analysis of the interaction effect between PVA and CGP on the released carbohydrates. Generally, linear increases in both polymers (X_1_X_2_) leads to a reduction in the content of released carbohydrates. However, linear increases in PVA concentration concomitant with quadratic increases of CGP (X_1_X_2_^2^) result in excess of unbound polysaccharides, increasing the amount of released carbohydrates.

**Figure 2:**
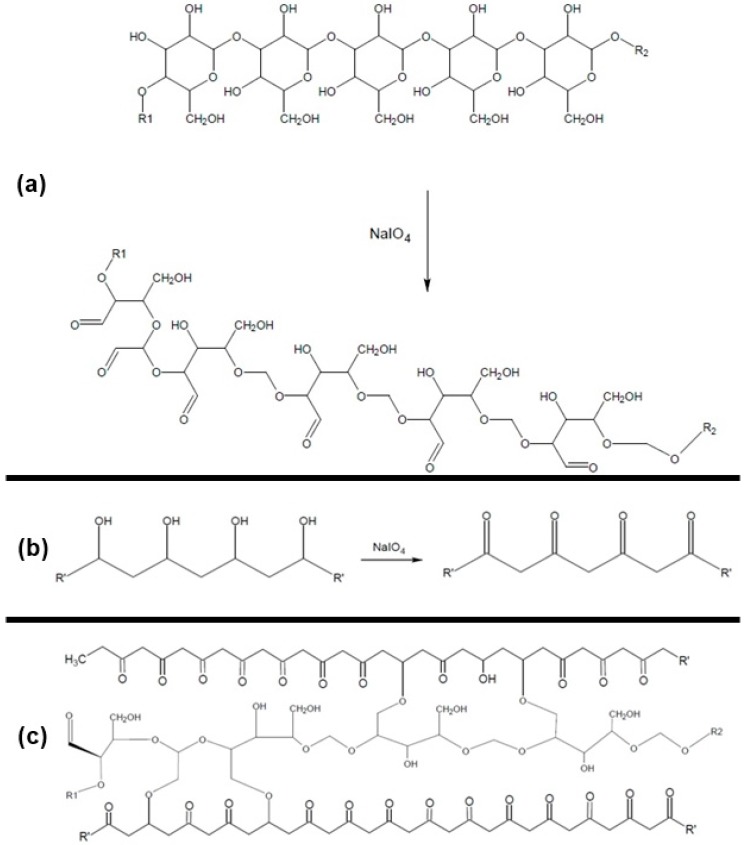
Scheme of (**a**) oxidative effect of oxidant agent (NaIO_4_) on the cashew gum polysaccharide (CGP) structure; (**b**) oxidative effect of NaIO_4_ on the polyvinyl alcohol (PVA) structure; and (**c**) chemical structure of the CGP/PVA films.

Finally, regarding the NaIO_4_ concentration (Figure 1), the positive linear effect (7.12) and the negative quadratic effect (−9.01) can be explained considering the oxidizing action of sodium periodate on the PVA and CGP. Periodate acts as oxidant on the vicinal hydroxyls of PVA generating reactive carbonyl groups (Figure 2b). Simultaneously, it promotes the oxidation of the vicinal hydroxyls (C_2_ and C_3_) on CGP, increasing the number of reactive groups available for chemical interactions with PVA (Figure 2a). Increases in the reactive groups in both polymers will increase the retention of CGP in the CGP/PVA network (Figure 2c), which explains the negative linear effect of NaIO_4_ in the released carbohydrates content.

However, according to Quan and Repeta [22], the exposure of polysaccharides to periodate under high temperatures and long reaction periods might lead to oxidation followed by hydrolysis of the glycosidic bonds. This action of periodate explains its negative quadratic effect. If the concentration of periodate is increased, the CGP chain is fragmented generating smaller fragments, which probably eliminates the steric hindrance which prevents these fragments to be incorporated in the network, increasing the content of released polysaccharides in the wash waters.

Additionally, the regression analysis for released carbohydrates showed an adequate fit of experimental values to a second-order polynomial model as a function of significant factors. The mathematical model is represented by Equation (2) (*r*^2^ = 0.96):

(2) $$y=139.79-3.23X_{1}^{2}-119.72X_{2}^{2}+35.57X_{2}-271.88X_{3}+196.16X_{3}^{2}+25.62X_{1}X_{2}-7.2X_{1}X_{2}^{2}$$

where *y* denotes the content of released carbohydrates (mg mL^−1^), *X*_1_, *X*_2_ and *X*_3_ denote the concentration of PVA, CGP and NaIO_4_, respectively. 

### 3.2. Swelling Index

Swelling is one of the most important properties of polysaccharide films, which characterize their use for biomedical applications [23,24,25]. The swelling index is a quantitative measure that represents the content of retained water into the polymer matrix, derived from interactions between the solvent molecules and the polar functional groups present in the chemical structure of the CGP/PVA components. Results from swelling studies are shown in Table 2. 

Multivariate analysis of the results from swelling index (SI) in the acidic environment of sodium acetate buffer (*r*^2^ = 0.84) revealed that the linear term of NaIO_4_ had the most pronounced effect on the response, negatively affecting the swelling index of the films. In addition, the quadratic term of PVA concentration and the interaction factor X_1_X_2_ also negatively affected the response. The quadratic term of CGP concentration was the only factor that positively affected the swelling index. 

Considering that the content of available polar groups in the polymer network interferes with the amount of interactions between the solvent molecules and the CGP/PVA film, increases in the concentration of NaIO_4_ in the formulation increases the number of binding sites between PVA and CGP, considerably decreasing the content of free hydroxyl groups in the PVA and CGP structures and enhancing the stiffness of the film structure. On the other hand, the three-dimensional structure of CGP contributed to the relaxation capacity of the polymer network in the presence of water and this is the reason why the increase of CGP in the formulation improves the swelling index.

**Figure 3:**
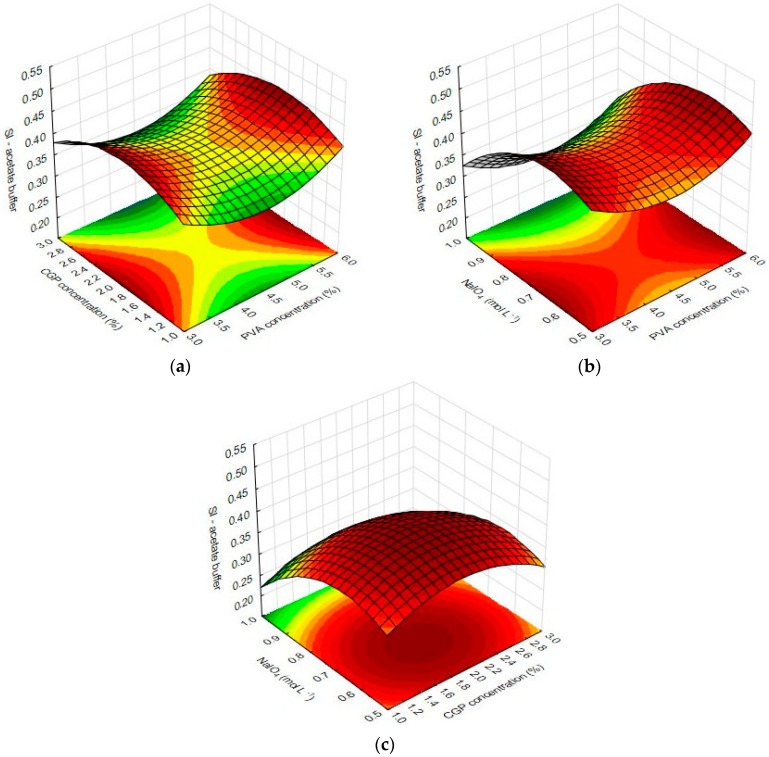
Response surface plots for swelling indexes of CGP/PVA films in acetate buffer as function of the significant factors: (**a**) effect of CGP and PVA concentrations; (**b**) effect of NaIO_4_ and PVA concentrations; and (**c**) effect of NaIO_4_ and CGP concentrations.

The graphical representation of significant effects of inter-relations and interactions of the independent variables on the swelling index of CGP/PVA films is depicted in Figure 3. As can be observed, there is a plateau of maximum values of swelling in CGP/PVA films produced with CGP around 2%, NaIO_4_ near 0.7 mol L^−1^ and PVA concentrations of 3% or 6%. 

Similar to that observed for the swelling index in the acetate buffer, results from multivariate analysis (ANOVA) indicate that the quadratic term of PVA negatively affected the swelling index (SI) in water, while the quadratic term of CGP positively affected this response. In addition, the multivariate analysis of SI in glycine buffer showed that PVA had the most pronounced effect on the swelling index, being observed as a negative effect of both linear and quadratic terms. Furthermore, the quadratic term of CGP positively affected the swelling index in the glycine buffer. It is noteworthy that the absence of the effect from NaIO_4_ changes from the acidic to a neutral or basic environment. In this case, the effect of NaIO_4_ on the network stiffness was overcome by the repulsion force between the carboxyl ionized groups from uronic acids present in the CGP structure. The ionization of these carboxyl groups is the main force responsible for the pH-stimuli responsive feature of the CGP/PVA films, as reported by Silva et al. [16]. In addition, despite the differences observed in the swelling behavior in the different solvents used, the values of swelling were similar to those obtained for other polysaccharide-based films [7,26,27,28]. 

### 3.3. Dimensional Changes

The three-dimensional variations during the swelling of CGP/PVA films were analyzed and results are shown in Table 2. As can be seen, the solvent used in the swelling test directly interfered with the three-dimensional behavior of CGP/PVA films, being observed with higher volume values for the films swollen in glycine buffer. 

Results from multivariate analysis showed that linear terms for PVA and CGP concentrations positively affected the values of volume for all tested solvents, whereas the content of NaIO_4_ just affected the responses for films swollen in acetate buffer and water, negatively interfering on the response. The quadratic terms for CGP and PVA did not influence the volume values for the films swollen in acetate buffer. On the other hand, the quadratic terms for CGP (−1.46) and PVA (−5.89) negatively affected the volume values for films swollen in glycine. For the films swollen in water, the quadratic term for CGP (4.31) positively affected the films volume while the quadratic term for PVA (−3.47) had a negative effect on this response.

The data from the volume of films swollen in the different solvents were converted into second-order polynomial equations. Consequently, the polynomial models describing the correlations between the response and the independent variables were presented as follows:

(3) $$y_{a}\left(cm^{3}\right)=0.20-0.12X_{2}-0.33X_{3}+0.04X_{2}^{2}+0.20X_{3}^{2}+0.03X_{1}X_{2}-0.01X_{1}X_{2}^{2}\;\left(r^{2}=0.97\right)$$

(4) $$y_{w}\left(cm^{3}\right)=0.30-0.05X_{1}-0.25X_{2}-0.22X_{3}+0.02X_{1}^{2}+0.07X_{2}^{2}+0.15X_{3}^{2}+0.06X_{1}X_{2}-0.02X_{1}X_{2}^{2}\;\left(r^{2}=0.98\right)$$

(5) $$y_{g}\left(cm^{3}\right)=\;0.17-0.04X_{1}-0.13X_{2}+0.002X_{1}^{2}+0.03X_{2}^{2}+0.03X_{1}X_{2}-0.01X_{1}X_{2}^{2}\;\;\left(r^{2}=0.98\right)$$

where *X*_1_, *X*_2_ and *X*_3_ denote the PVA, CGP and NaIO_4_ concentrations, *y_a_*, *y_w_* and *y*_g_ denote the three-dimensional behavior of films swollen in acetate buffer, water and glycine buffer, respectively. The fitness was expressed by the *r*^2^ values, which indicates that more than 97% of the variability in the response can be explained by the models (Equations (3)–(5)). This suggests that the models represented accurately the data in the experimental region.

**Figure 4:**
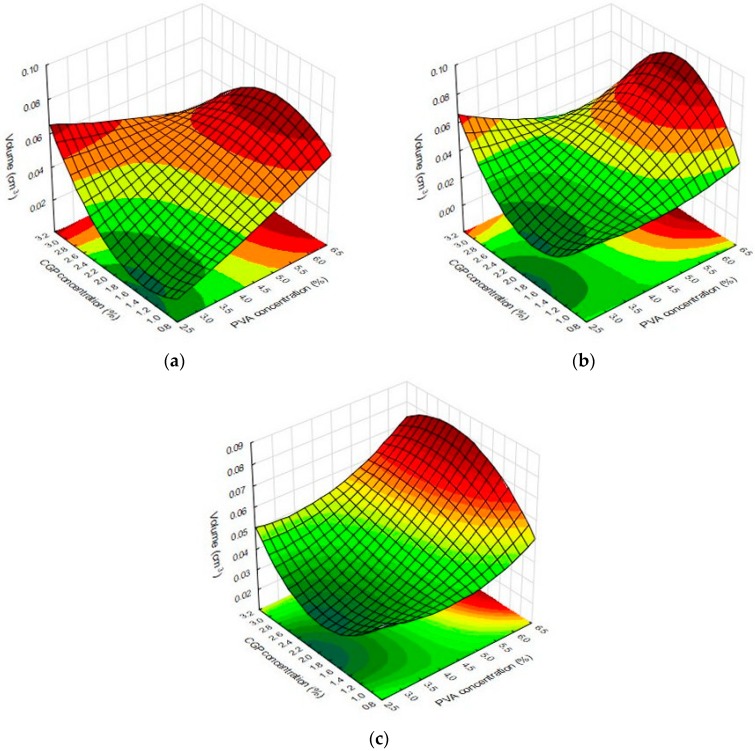
Response surface plots for the dimension changes of CGP/PVA films in (**a**) acetate buffer, (**b**) water and (**c**) glycine buffer as function of the significant factors.

Using the response surface methodology, the interaction and inter-relations of the independent variables were analyzed and the results are depicted in Figure 4. As can be observed, the three-dimensional profile of the films swollen in water (Figure 4b) and glycine buffer (Figure 4c) were quite similar, showing higher values of volume in the films containing a higher PVA concentration and CGP concentration of about 2% For the dimensional changes occasioned by swollen films in the sodium acetate buffer, it was observed that a plateau of maximum values in the films produced with CGP concentration higher than 2.4% and PVA concentration above 5% (Figure 4a). These results can be explained since films with more mass are expected to present higher expansion in the three-dimensional area. 

### 3.4. Microstructural Characterization of the CGP/PVA Films

**Figure 5:**
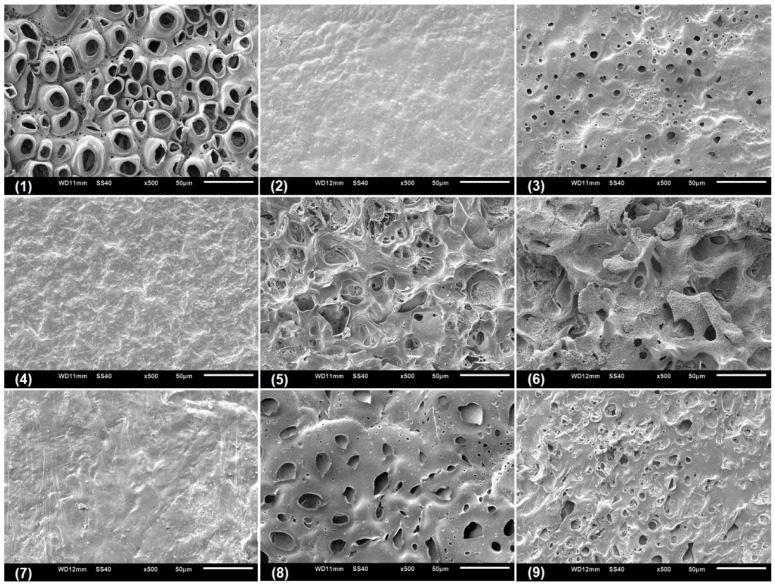
Scanning electron micrographs of CGP/PVA films swollen in acetate buffer (pH 4.5).

**Figure 6:**
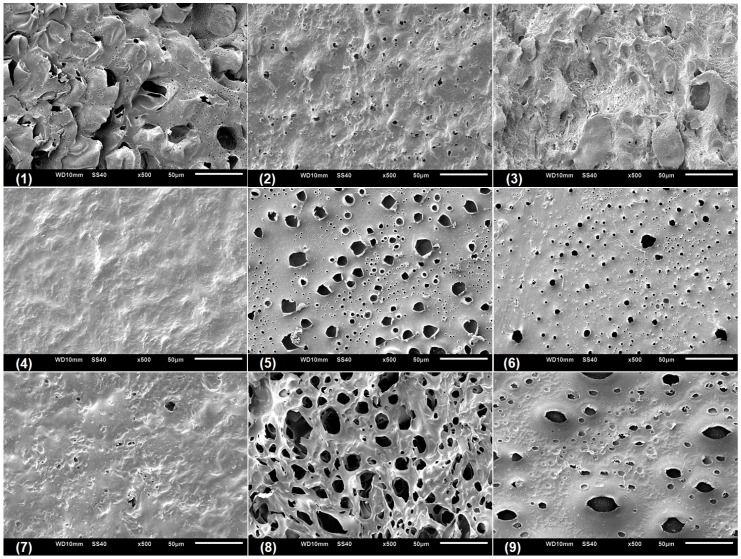
Scanning electron micrographs of CGP/PVA films swollen in ultrapure water.

**Figure 7:**
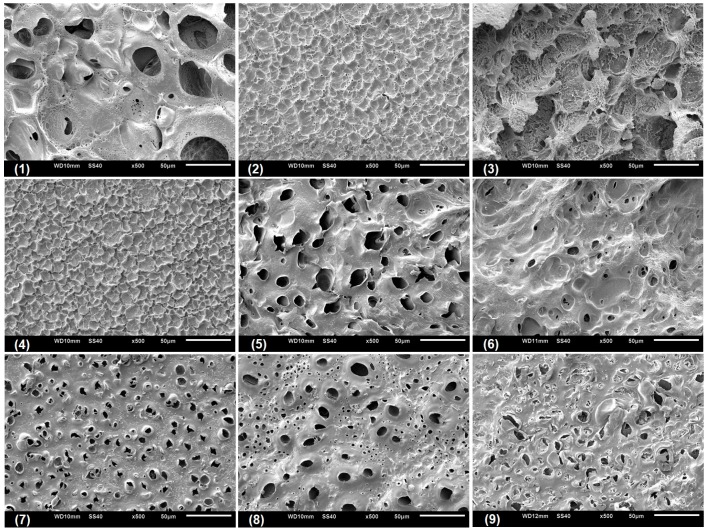
Scanning electron micrographs of CGP/PVA films swollen in glycine buffer (pH 9.5).

Scanning electronic microscopy (SEM) was performed with the aim to analyze the microstructural characteristic of the swollen CGP/PVA films (Figure 5, Figure 6 and Figure 7). As can be seen, CGP/PVA films can be roughly divided in three distinct groups: (1) Films with a wide-ranging of pores incidence (films (1) (5), (6), (8) and (9) from Figure 5, Figure 6 and Figure 7); (2) films where the presence of pores is not well defined (films (3) and (7) from Figure 5, Figure 6 and Figure 7); and (3) films without pores (films (2) and (4) from Figure 5, Figure 6 and Figure 7). 

SEM micrographs evidence that the film constituents are quite important to determine the structural characteristics of the network. In addition, it is possible to infer that the concentration of NaIO_4_ is of major importance in the pore size and shape. In general, in the formulations produced with 1.0 mol L^−1^ NaIO_4_ there was a total absence of pores, independent of the swelling agent (films (2) and (4) from Figure 5, Figure 6 and Figure 7). However, the presence of pores in the film for Run 9 (Figure 5, Figure 6 and Figure 7) can be related to the higher content of polymers, which allowed a higher relaxation of the matrix and consequently contributes to the pore formation.

For the films produced with NaIO_4_ at concentrations of 0.75 mol L^−1^ or 0.5 mol L^−1^, the SEM analysis evidenced the presence of pores differing in incidence and size. In these formulations, the polymer concentration seems to influence the degree of porosity (Figure 5, Figure 6 and Figure 7). In general, swelling in the acetate buffer resulted in materials with lower pore distribution and size except for Run 1 (Table 3). The higher number of pores with size above 10 μm for the films produced in Run 1 can be related to a combined effect of lower content of polymers and oxidant agent, which makes the CGP/PVA matrix less cohesive and favored the interactions with the solvents during swelling (Table 3). Furthermore, it was observed that there was a higher morphologic diversity in the films swollen in water and glycine buffer (Figure 6 and Figure 7, Table 3).

### 3.5. Light Transmission Properties

When a certain material presents the optical property of light transmission higher than its light absorptivity and reflectivity, the material is classified as a transparent material. Materials classified as transparent media have several applications in a diversity of economic fields [29]. The regular light transmission profiles of CGP/PVA films before and after swelling are shown in Table 4. As can be observed, the dried films showed light transmission values ranging from 18.2% to 74.6% depending on their composition. Nevertheless, the swelling process drastically improved the light transmission profile of the films, being observed as transmission values varying from 37.2% to 91.4%. In general, wet films containing a higher CGP and PVA concentration showed the highest values of light transmission, presenting values above 70%. 

The multivariate analysis results indicate that only the linear terms for PVA (X_1_) and CGP (X_2_) affected the light transmission values for both dried and wet films. However, the magnitude of factors interference was completely opposite, being observed a negative effect on the response for the dried films and a positive effect for the swollen films. Positive effects of CGP and PVA concentrations for the swollen films can be explained due to their interaction with water molecules through hydrogen bonds, increasing the amount of water inside the film and consequently decreasing the resistance to pass light throughout. On the other hand, in the dried state the chains of CGP and PVA will represent a physical barrier to the light passage, and then increasing their concentration in the film formulation will negatively affect the light transmission. 

Moreover, the values of light transmission of the CGP/PVA films allow a multitude of biotechnological applications for this material, since it does not compromise the visualization of the product, an essential parameter for commercialization.

### 3.6. Thickness

Thickness is a physical property defined as the perpendicular distance between two surfaces. This parameter has a high importance for characterizing mechanical properties of both conventional plastics and polysaccharide-based films, affecting aspects such as uniformity, homogeneity and reproducibility [30,31,32]. The thickness will depend on the ratio between the mold area and the volume/mass of film-forming solution. In this study, the volume/area ratio was kept constant (1 mL cm^−2^), but there were variations in the ratio between the area and mass of material placed in the molds for polymerization. Moreover, after polymerization, films were submitted to washes for removal of unbound polysaccharides, which may have affected the amount of material retained in the film network. In general, the thickness values of the produced CGP/PVA films were similar to those from commercial chitosan-starch films [33] and lower than those from cellulose/acetate/starch [34] and cellulose/polymethacrylate blended films [26].

Results from thickness measurements shown in Table 4 evidenced that CGP/PVA films from Run 7 and 8 presented the highest values of thickness, a predictable result since they were produced employing the highest PVA concentration (6%) and CGP concentrations of 1% (Run 7) and 2% (Run 8). However, the film from Run 9, produced using the highest concentrations of both PVA (6%) and CGP (3%), obtained values of thickness lower than those presented in Run 7 and 8, which might be explained by the higher content of carbohydrates leached from CGP/PVA films produced in Run 9 when compared to those produced in Run 7 and Run 8 (Table 2).

Thickness is a parameter that interferes in the light transmission and therefore, can alter the transparency of CGP/PVA films. Thicker films increase the absorption rate of the incident light, decreasing the transmittance detected in the spectrophotometer. Thus, we performed a Pearson’s correlation analysis between thickness values and light transmission values. Results showed a weak correlation between these parameters (*r* = 0.46) in the range of thickness studied for CGP/PVA films. Therefore, the composition factors (CGP, PVA and NaIO_4_ concentration) have a more pronounced effect on the light transmission of the films than thickness. In fact, the action of NaIO_4_ in reducing the capacity of the matrix to interact with water in the medium decrease the light transmission of the films and strongly interfere in the film transparency (*r* = 0.99) for wet films, whereas the CGP and PVA concentration are the main factors influencing the dried film’s transparency.

### 3.7. Mechanical Properties

Mechanical properties of films reflect the behavior of the material to an applied force and are dependent of the inter- and intramolecular interactions between the components of the polymeric material [35]. In this work, the produced CGP/PVA films were evaluated regarding their traction force and elongation properties (Table 4). The traction force (TF) at rupture, is a measure represented by the maximum force offered to a specimen at the rupture point during extension under traction. The elongation (E) can be related to the elasticity of a material since it is measured by the extension under traction [16].

The multivariate analysis of data from the traction force showed a significant effect of NaIO_4_ and CGP on the response. The linear term for NaIO_4_ had the most pronounced effect (79.99) on this response. The quadratic term for the CGP concentration also positively affected (55.66) the traction force, while the quadratic term for NaIO_4_ had a negative effect on this response (−37.13). 

**Figure 8:**
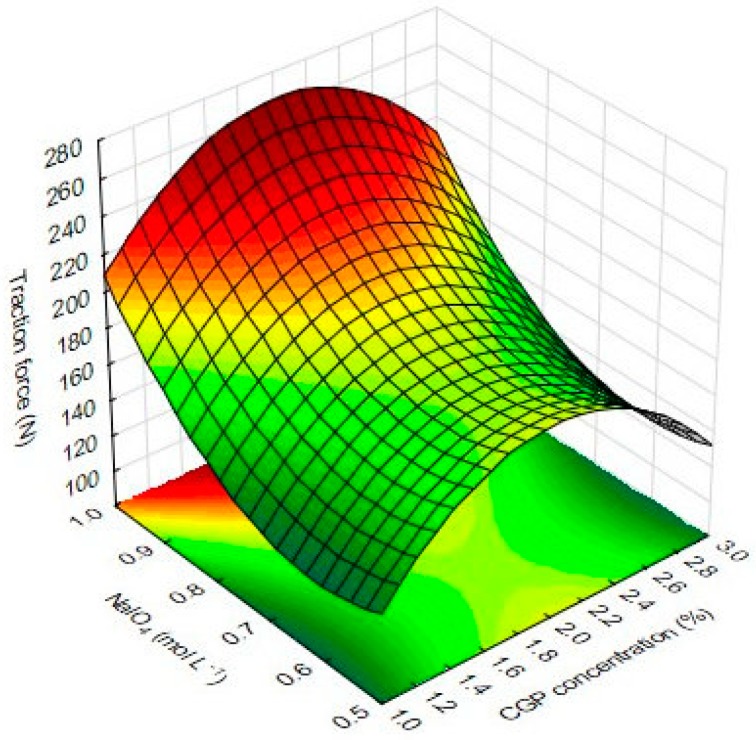
Response surface plots for traction force results of CGP/PVA films as function of the significant factors.

The effects of inter-relations and interactions of the independent variables on the traction force CGP/PVA films are depicted in Figure 8. 

The diagram describes the variations on the response as a function of statistically significant components. The response surface plot for TF considering the effect of NaIO_4_ and CGP showed an overall curvilinear profile (Figure 8). As can be observed, increases in the concentration of NaIO_4_ leads to increases in the TF values, which can be a result of its oxidative effect in the CGP by increasing the number of reactive sites able to interact with PVA. Thus, the resulting material presented a higher traction force under stretching. On the other hand, NaIO_4_ also showed a negative quadratic effect, evidencing that in high concentrations and/or high reaction time, this factor can cause the hydrolysis of CGP glycosidic bonds [10,22], minimizing the number of binding sites with PVA, and thus, causing a decrease in TF values (Figure 8).

Regarding to the elasticity tests (Table 4), the multivariate analysis (*r*^2^ = 0.99) showed that only the linear term for PVA concentration affected the elasticity of CGP/PVA films, positively interfering (4.54) on this response. The results can be explained by the conformational structure of the PVA, which is a long linear chain being able to distend along its own axis. This ability enables the polymer matrix to extend without brake when submitted to considerable high traction forces values.

Comparing the mechanical properties of CGP/PVA films with literature data, it was observed that the obtained films presented similar EB values and TF values higher than those obtained for polyolefinic and starch-based films [27]. In addition, the mechanical properties of CGP/PVA were also higher than those for other protein and polysaccharide-based films [28,36,37], indicating that these materials have interesting properties, which allows a multitude of applications of CGP/PVA films. 

## 4. Conclusions 

Nowadays, biomaterials produced by blending natural polysaccharides with biodegradable synthetic polymers such as polyvinyl alcohol have been exhaustively tested as substitutes for petrochemical-based films [38,39,40,41,42]. In this scenario, CGP/PVA films are quite interesting since besides their interesting physicochemical and mechanical properties, they present 50% degradation after 90 days in soil-burial tests [43]. In addition, considering the morphological, optical and mechanical properties of the produced CGP/PVA films it is possible to suggest the utilization of these materials in different areas. 

The high values of light transmission and tensile strength suggest that CGP/PVA films have a great potential of utilization as food packaging, especially those formulations with a higher content of PVA and CGP (Run 8 and 9), who presented tensile strength values much higher than those reported to synthetic polymers such as polyethylene and polystyrene [44,45,46,47]. Furthermore, CGP/PVA films from Runs 4–9 (Table 4) have superior mechanical properties when compared to those of several other polysaccharide-based films. The produced film also has as attractive high values of elongation, which guarantee a lower fragility and consequently improves their performance as covering materials [47,48,49,50,51,52]. In addition, these films are highly stable during storage, maintaining these mechanical and physicochemical properties after years of storage at room temperature. 

The development of biodegradable materials has also received great attention in the biomedical area, where alternative biodegradable and biocompatible scaffold materials and drug carries are extensively studied [53,54]. The CGP/PVA films herein produced have morphological characteristics that enable their use as both scaffold and carrier materials. The microstructure of films produced in Run 1, 3, 5 and 8 (Figure 5, Figure 6 and Figure 7) evidences that they can act as three-dimensional supports for cell attachment and subsequent tissue organization and formation. Furthermore, CGP/PVA films produced in Run 7 showed an excellent pH-responsive behavior (Figure 5, Figure 6 and Figure 7), changing from a non-porous structure in acidic medium to a highly porous profile in a basic environment (Table 3). This feature enables the use of these films as smart drug delivery materials. In addition, the varied microstructure profiles of the produced CGP/PVA films allow their application as immobilization supports of a wide range of biomolecules, with different molecular weight or chemical features.

In summary, this work showed that using a 3^3-1^ fractional factorial design it was possible to distinguish the effect of every component of the CGP/PVA film formulations on the chemical, physical and mechanical properties of the produced materials. First, we can determine the optimal proportions of polymers and oxidant agent to avoid waste of material. Second, the determination of the role of every component in the film formulation allows proposing the right formulation for the desired application, predicting the property of the synthesized material, what is in consonance with the consumer and market needs. Finally, considering their biodegradability and biocompatibility features, the CGP/PVA films produced in this work can be used as a renewable and eco-friendly material in substitution of the current petrochemical-based films.

## Figures and Tables

**Table 1:** Factors coded (in parenthesis) and decoded levels used in the 3^3-1^ fractional factorial design to produce CGP/PVA films.

Run	PVA (X_1_)	CGP (X_2_)	NaIO_4_ (X_3_)
1	3.0 (−) ^1^	1.0 (−) ^1^	0.5 (−) ^2^
2	3.0 (−)	2.0 (0)	1.0 (+)
3	3.0 (−)	3.0 (+)	0.75 (0)
4	4.5 (0)	1.0 (−)	1.0 (+)
5	4.5 (0)	2.0 (0)	0.75 (0)
6	4.5 (0)	3.0 (+)	0.5 (−)
7	6.0 (+)	1.0 (−)	0.75 (0)
8	6.0 (+)	2.0 (0)	0.5 (−)
9	6.0 (+)	3.0 (+)	1.0 (+)

^1^ Values are referred to the percentage of polymer. ^2^ Values are referred as concentration, in mol L^−1^.

**Table 2:** Results for the content of released carbohydrates, swelling properties and dimensional changes from CGP/PVA films produced in the 3^3-1^ fractional factorial design.

Run	PVA	CGP	NaIO_4_	RC ^1^	SI_A_^2^	SI_W_^3^	SI_G_^4^	DC_A_^5^	DC_w_^6^	DC_G_^7^
1	3.0 (−) ^1^	1.0 (−) ^1^	0.5 (−) ^2^	22.3	0.35	0.38	0.48	0.035	0.033	0.038
2	3.0 (−)	2.0 (0)	1.0 (+)	32.4	0.35	0.34	0.48	0.025	0.025	0.027
3	3.0 (−)	3.0 (+)	0.75 (0)	41.2	0.38	0.35	0.48	0.051	0.047	0.043
4	4.5 (0)	1.0 (−)	1.0 (+)	38.5	0.22	0.22	0.25	0.039	0.042	0.038
5	4.5 (0)	2.0 (0)	0.75 (0)	25.5	0.36	0.33	0.35	0.039	0.042	0.043
6	4.5 (0)	3.0 (+)	0.5 (−)	43.2	0.33	0.23	0.25	0.066	0.050	0.053
7	6.0 (+)	1.0 (−)	0.75 (0)	11.2	0.43	0.36	0.28	0.055	0.049	0.052
8	6.0 (+)	2.0 (0)	0.5 (−)	28.7	0.43	0.40	0.38	0.081	0.085	0.070
9	6.0 (+)	3.0 (+)	1.0 (+)	35.1	0.24	0.25	0.29	0.051	0.053	0.067

^1^ Release carbohydrates (mg mL^−1^). ^2^ Swelling indexes in acetate buffer. ^3^ Swelling indexes in water. ^4^ Swelling indexes in glycine buffer. ^5^ Dimensional changes in acetate buffer (cm^3^). ^6^ Dimensional changes in water (cm^3^). ^7^ Dimensional changes in glycine buffer (cm^3^).

**Table 3:** Distribution and relative frequencies for pore size of each CGP/PVA film produced using the 3^3-1^ fractional factorial design.

Run	Pore Size	Relative Frequency (%)		
		Acetate Buffer	Water	Glycine Buffer
1	0–5	7.9	50.0	32.0
	5–10	16.0	14.6	6.3
	10–20	65.6	22.2	26.0
	20–30	9.0	5.8	22.0
	>30	1.5	7.4	13.7
2	0–5	n.d. ^1^	90.8	100
	5–10	n.d.	9.2	n.d.
3	0–5	76.3	92.7	n.d.
	5–10	23.7	7.3	n.d.
4	0–5	n.d.	n.d.	100
5	0–5	44.2	90.8	5.7
	5–10	27.4	8.0	13.5
	10–20	17.9	1.2	60.4
	20–30	6.3	n.d.	17.3
	>30	4.2	n.d.	3.1
6	0–5	55	100	76.8
	5–10	18.8	n.d.	23.2
	10–20	22.5	n.d.	n.d.
	20–30	1.2	n.d.	n.d.
	>30	2.5	n.d.	n.d.
7	0–5	n.d.	100	54.2
	5–10	n.d.	n.d.	45.8
8	0–5	40.6	20.6	69.5
	5–10	18.1	33.3	8.0
	10–20	34.9	45.1	20.5
	20–30	6.4	1.0	2.0
9	0–5	71.6	20.6	59.5
	5–10	20.3	25.4	29.8
	10–20	7.2	23.8	10.7
	20–30	0.9	11.2	n.d.
	>30	n.d.	19.0	n.d.

^1^ not detected.

**Table 4:** Results for thickness, optical and mechanical properties from CGP/PVA films produced using the 3^3-1^ fractional factorial design.

Run	Thickness ^1^ (μm)	Light Transmission (%)		Mechanical Properties ^1^	
		Dried Film	Swollen Film	Traction Force (n)	Elongation (mm)
1	246	73.4	39.4	106.3	10.33
2	199	68.1	47.7	252.8	10.66
3	164	61.4	70.4	112.5	11.27
4	327	74.6	32.7	205.9	23.50
5	357	65.8	61.6	185.9	35.29
6	383	37.6	86.1	138.2	24.16
7	517	69.6	57.3	127.5	26.40
8	528	22.3	81.8	172.8	40.23
9	423	18.2	91.4	198.6	37.88

^1^ Results are means of 10 measurements.
